# Matrix Interference Removal Using Fe_3_O_4_@SiO_2_-PSA-Based Magnetic Dispersive Solid-Phase Extraction for UPLC-MS/MS Analysis of Diazepam in Aquatic Products

**DOI:** 10.3390/foods14142421

**Published:** 2025-07-09

**Authors:** Mengqiong Yang, Guangming Mei, Daoxiang Huang, Xiaojun Zhang, Pengfei He, Si Chen

**Affiliations:** 1 Food and Pharmacy College, Zhejiang Ocean University, Zhoushan 316022, China; ymengqiong@163.com (M.Y.);; 2 Zhejiang Marine Fisheries Research Institute, Zhoushan 316021, China

**Keywords:** aquatic products, benzodiazepine, veterinary drugs, food safety, UPLC-MS/MS

## Abstract

A sensitive method was developed for detecting diazepam residues in aquatic products using magnetic dispersive solid-phase extraction (MDSPE) coupled with ultra-performance liquid chromatography–tandem mass spectrometry (UPLC-MS/MS). Samples extracted with 1% ammonia–acetonitrile were purified using synthesized Fe_3_O_4_@SiO_2_-PSA nanoparticles via MDSPE before UPLC-MS/MS analysis. Separation was performed on a C_18_ column with gradient elution using 0.1% formic acid–2 mM ammonium acetate/methanol. Detection employed positive electrospray ionization (ESI^+^) in multiple reaction monitoring (MRM) mode. Characterization confirmed Fe_3_O_4_@SiO_2_-PSA’s mesoporous structure with excellent adsorption capacity and magnetic properties. The method showed good linearity (0.1–10 μg/L, *r* > 0.99) with an LOD and LOQ of 0.20 μg/kg and 0.50 μg/kg, respectively. Recoveries at 0.5–15.0 µg/kg spiking levels were 74.9–109% (RSDs 1.24–11.6%). This approach provides rapid, accurate, and high-precision analysis of diazepam in aquatic products, meeting regulatory requirements.

## 1. Introduction

Diazepam, commonly known as Valium, belongs to the benzodiazepine class of sedative-hypnotics and is a class II psychotropic controlled substance. It is widely used in the treatment of anxiety, sedation, insomnia, convulsions, epilepsy, central muscle relaxation, and other disorders. Since its development in the 1950s, diazepam has gradually become one of the most successful and influential drugs in psychopharmacology [1]. Diazepam can increase meat yield in food animals, reduce animal mobility, and alleviate stress, which leads to its illegal use in animal farming [2]. In recent years, diazepam residues have been reported across different regions of China and in a variety of aquatic products, such as *Litopenaeus vannamei* [3], *Ctenopharyngodon idella* [4], and *Carassius auratus* [5]. Diazepam has difficulty being metabolized by animal tissue, leading to its accumulation in food animals. It can enter the soil and water through animal excreta, causing ecological problems. Meanwhile, long-term intake of food containing diazepam and its metabolite residues is detrimental to human health, hinders the sustainable development of the aquaculture industry, and poses a serious threat to food safety [6]. Therefore, rapid and efficient detection methods have become necessary for monitoring diazepam residues in foods of animal origin.

Rapid and accurate quantification of trace targets in complex aquatic products is a challenging problem; therefore, adsorption and cleanup of targets through sample pretreatment are required to reduce matrix interference before quantitative analysis [7,8]. Currently, the main pretreatment methods for the detection of diazepam residues in biological samples are liquid–liquid extraction (LLE) [9,10], solid-phase extraction (SPE) [11], dispersive solid-phase extraction (DSPE) [12], molecularly imprinted solid-phase extraction (MISPE) [13], and magnetic solid-phase extraction (MSPE) [14,15]. MSPE is a new pretreatment technology that combines the advantages of magnetic separation and SPE, and stands out among many improved SPE methods. Nanoparticles (NPs), as new nanomaterials with unique properties [16], are ideal SPE adsorbents and have been widely used in various fields, including pesticide residues [17], antibiotics [18], textiles [19], polycyclic aromatic hydrocarbons [20], endocrine-disrupting compounds [21], toxins [22], metals [23], and proteins [24]. Currently, the detection of diazepam residues in aquatic products faces the following challenges: (1) the sample matrix is complex, with considerable interference from proteins and lipids; (2) the metabolites are structurally diverse, which makes it difficult to achieve simultaneous detection using traditional methods; and (3) the existing SPE methods suffer from drawbacks such as high consumption of organic solvents and cumbersome operation. Since Paul Anastas proposed the 12 principles of green chemistry in 1990, environment-friendly sample pretreatment technology has become a research hotspot in the field of analytical chemistry [25]. MSPE, as the third generation of green separation technology, has significantly replaced the traditional organic solvent extraction methods. MSPE allows easy separation, and the adsorbent can be isolated from the solution in a short time by applying a magnetic field, thereby eliminating the need for centrifugation or filtration steps used in traditional SPE. Thus, the magnetic adsorbent is easy to recover and regenerate, reducing reagent consumption and waste generation. In summary, MSPE has the advantages of high efficiency, rapidity, simplicity, high selectivity, and environmental friendliness, and shows great potential in the pretreatment of complex matrices. However, the selective adsorption of diazepam-like substances using conventional magnetic materials is insufficient [26], and there is an urgent need to develop new functionalized magnetic adsorbents.

Currently, the main methods for the detection of diazepam in biological samples include biosensors [27], colloidal gold-based assay [28], enzyme-linked immunosorbent assay [29], gas chromatography–mass spectrometry [30], and liquid chromatography–mass spectrometry (LC-MS) [31]. The coupling of high-performance liquid chromatography (HPLC) and MS allows for high-resolution separation and high-sensitivity detection, which is capable of accurately identifying and quantitatively analyzing trace components in complex samples. This technique has become the mainstream method for determining veterinary drug residues in food. In this study, a magnetic nanoparticle material Fe_3_O_4_@SiO_2_-PSA was newly synthesized and applied as an MSPE adsorbent for matrix interference removal during aquatic sample purification. Unlike conventional MSPE targeting analytes, this strategy eliminated centrifugation and desorption, enhancing efficiency, reducing reagent use, and enabling rapid cleanup. Coupled with UPLC-MS/MS, it established a highly sensitive and selective method for determining diazepam residue in aquatic products, providing a new and reliable monitoring approach.

## 2. Materials and Methods

### 2.1. Reagents and Materials

Diazepam standard solution (methanol medium) with a mass concentration of 1.0 mg/mL was obtained from TanMo Reference Materials Co., Ltd. (Changzhou, China). Formic acid, methanol, acetonitrile, and ammonium acetate (chromatographic-grade) were purchased from Merck KGaA (Darmstadt, Germany). Anhydrous MgSO_4_, sodium citrate, ethylene glycol, ethanol, cetyltrimethylammonium bromide, 25–30% concentrated ammonia, tetraethyl orthosilicate, acetone, toluene, and diethylamine (analytical-grade) were procured from Sinopharm Chemical Reagent Co., Ltd. (Shanghai, China). Analytical-grade ferric chloride and N,N-diethyl-3-(trimethoxysilyl)propylamine (PSA) were obtained from Shanghai Aladdin Biochemical Technology Co., Ltd. (Shanghai, China). Ultrapure water used in the experiments was prepared using a Milli-Q Advantage A10 system (Millipore, Billerica, MA, USA).

### 2.2. Instruments and Equipment

An Acquity UPLC I-class ultra-high-performance liquid chromatography coupled with a Xevo TQ-S triple quadrupole mass spectrometer equipped with an electrospray ionization (ESI) ion source (Waters, Milford, MA, USA) was used for chromatographic and mass spectrometric analysis. Sample mixing and magnetic stirring were carried out using an MS3D vortex mixer (IKA, Staufen, Germany) and a DF-101S magnetic stirrer (Bangxi Instrument Technology Co., Ltd., Shanghai, China), respectively. Centrifugation was performed using a benchtop centrifuge 5810 (Eppendorf, Hamburg, Germany), while homogenization was conducted using an FJ200-SH high-speed homogenizer (Hangzhou Jingfei Instrument Technology Co., Ltd., Hangzhou, China). Sonication was performed using an FS-2000T ultrasonic processor (Shanghai Shengxi Supersonic Instrument Co., Ltd., Shanghai, China). Characterization instruments included a JEM-2100F transmission electron microscope (JEOL, Tokyo, Japan), a Geminisem 300 scanning electron microscope (Carl Zeiss AG, Baden-Württemberg, Germany), a Miniflex 600 X-ray diffractometer (Rigaku, Tokyo, Japan), and a Fisher Scientific Nicolet iS5 Fourier transform infrared spectroscopy (FTIR) spectrometer (Thermo Fisher, Waltham, MA, USA). Magnetic properties were analyzed using a LakeShore 7404 vibrating sample magnetometer (LakeShore, Cleaveland, OH, USA), and surface area and porosity were determined using an ASAP 2460 automatic specific surface and porosity analyzer (Micromeritics, Norcross, GA, USA).

### 2.3. Experimental Methods

#### 2.3.1. Preparation of Standard Solutions

Diazepam standard stock solution: 1 mL diazepam standard solution was accurately pipetted and diluted to 100 mL with methanol to obtain a diazepam standard stock solution at a concentration of 10 µg/mL. This solution was stored at −20 °C refrigerator in the dark with a storage shelf-life of 12 months.

Diazepam standard working solution: 100 µL of the diazepam standard stock solution was taken and then diluted to 10 mL with methanol to prepare a working solution having a concentration of 100 ng/mL. It was then stored in a refrigerator at −20 °C in the dark, with a validity period of 3 months.

Blank matrix-matched calibration solutions: with volumes of 10, 25, 50, 200, 500, and 1000 μL were accurately aspirated from the diazepam standard working solution into separate tubes, and the final volume was made to 10 mL with the blank matrix extract solution (prepared by extracting the negative samples using the method of “pre-treatment of samples” in Section 2.3.3). These solutions were mixed and then filtered through a 0.22 μm membrane, which was then used to prepare a series of calibration standard solutions with mass concentrations of 0.1, 0.25, 0.5, 2.0, 5.0, and 10.0 ng/mL, respectively.

#### 2.3.2. Preparation and Structural Characterization of Magnetic NPs Fe_3_O_4_@SiO_2_-PSA

Preparation of Magnetic NPs Fe_3_O_4_@SiO_2_-PSA

Preparation of Fe_3_O_4_: 0.4 g of sodium citrate was dissolved in 72 mL of ethylene glycol. As an organic ligand, sodium citrate contains carboxylate ions (-COO-), which facilitates the subsequent grafting of functional molecules to fabricate core–shell structures. Next, 16 mL of solution A (5.0 g of FeCl_3_-6H_2_O was dissolved in 100 mL of ethylene glycol) was added slowly, followed by solution B (10.0 g of sodium acetate was dissolved in 72 mL of ethylene glycol). The mixture was stirred vigorously at 600 rpm using a magnetic stirrer for 30 min. It was then transferred into a 200 mL PTFE-lined stainless steel reactor and solvent-heated at 180 °C for 10 h. After the reaction system was cooled naturally to room temperature, the products were separated using an applied magnetic field, washed with deionized water and anhydrous ethanol thrice to remove organic residues, and finally dried under vacuum for 12 h at 60 °C to obtain magnetic Fe_3_O_4_ NPs.

Preparation of Fe_3_O_4_@SiO_2_: First, 1.0 g of Fe_3_O_4_ NPs was dispersed in 100 mL of pure water and ultrasonicated for 30 min to form a homogeneous suspension. This suspension was transferred to a 500 mL three-necked flask preloaded with 2.0 g of hexadecyltrimethylammonium bromide and 200 mL of pure water and then injected with 65 µL of concentrated ammonia and purged with nitrogen gas for protection. Under the magnetic stirring at 600 rpm, 2.5 mL of tetraethyl orthosilicate and 1.4 mL of anhydrous ethanol were added sequentially, and the reaction was continued at 60 °C for 12 h. At the end of the reaction, solid–liquid separation was carried out by applying a magnetic field, and the product was washed thrice with acetone and anhydrous ethanol sequentially, and finally dried at 60 °C for 12 h in a vacuum drying oven to obtain core-shell Fe_3_O_4_@SiO_2_ NPs.

Preparation of Fe_3_O_4_@SiO_2_-PSA: 0.6 g of core–shell Fe_3_O_4_@SiO_2_ NPs and 250 mL of anhydrous toluene were added to a 500 mL three-necked flask. Next, 3 mL of PSA, 3 mL of anhydrous toluene, and 1 mL of diethylamine were added sequentially under nitrogen gas protection and stirred magnetically for 30 min at room temperature at 600 rpm. The reaction was magnetically stirred at 600 rpm for 30 min at room temperature, and then heated to 65 °C for 6 h. After the completion of the reaction, the products were separated using an applied magnetic field, washed sequentially with acetone, anhydrous ethanol, and deionized water thrice, and finally dried at 60 °C for 12 h under vacuum to obtain Fe_3_O_4_@SiO_2_-PSA magnetic NPs functionalized with surface amino groups.

Structural Characterization of Fe_3_O_4_@SiO_2_-PSA

Two milligrams of the sample were dispersed in ethanol solution and sonicated for 20 min. A 4 μL aliquot of the suspension was added dropwise to a copper mesh support film, dried at room temperature, and then subjected to transmission electron microscopy (TEM) analysis at an accelerating voltage of 60 kV. Another two milligrams of the sample was dispersed in 2 mL ethanol, sonicated for 5 min, and then added dropwise to an aluminum sample stage coated with a 5 nm gold film for scanning electron microscopy (SEM) analysis at an accelerating voltage of 5–10 kV. X-ray diffraction (XRD) analysis was carried out after the sample powder was evenly spread onto the center of the slide using a CuKa radiation source (λ = 0.15405 nm), with a scanning range of 5–80° and a scanning rate of 4°/min. The samples were mixed with dried potassium bromide powder at 1:100 (*w*/*w*) and ground to homogeneity in an agate mortar, pressed into transparent flakes, and then subjected to FTIR spectroscopy over a scanning range of 4000–400 cm^−1^, with a resolution of 4 cm^−1^ and a cumulative total of 64 scans. The samples were wrapped with paraffin film and fixed onto a vibrating sample rod before being subjected to a vibrating sample magnetometer (VSM) analysis at room temperature (300 K) under a magnetic field in the range of ±20 kOe. For the Brunauer–Emmet–Teller (BET) test, the samples were degassed at 120 °C for 6 h under a nitrogen atmosphere to remove surface adsorbates. The nitrogen adsorption–desorption isotherm was subsequently determined at the temperature of liquid nitrogen (77 K), with relative pressures (P/P_0_) ranging from 0.05 to 1.00.

#### 2.3.3. Sample Processing and Analysis

Pre-Treatment of Samples

The aquatic products used in the experiment, such as *Carassius auratus*, *Litopenaeus vannamei*, *Portunus trituberculatus*, and *Mytilus edulis*, were purchased from Fengmao Farmer’s Market in Lincheng, Zhoushan City, Zhejiang Province, China. The edible part of the samples was collected after removing the shells, viscera, or bones, homogenized, and then stored in the refrigerator at −18 °C for freezing. A 5.00 g thawed sample was weighed into a 50 mL centrifuge tube. Then, 15 mL of acetonitrile solution containing 1% ammonia was added and the mixture was vortexed for 1 min, followed by the addition of 5 g MgSO_4_ and 1.25 g NaCl. The mixture was again vortexed for 3 min and centrifuged at 5000 rpm for 3 min. Subsequently, 2 mL of the upper layer of acetonitrile was transferred into a 2 mL centrifuge tube containing 30 mg Fe_3_O_4_@SiO_2_-PSA and 30 mg of C_18_. This mixture was vortexed and shaken for 3 min, and the magnetic adsorbent was then rapidly separated under an external magnetic field. Finally, 1.0 mL of the purified supernatant was filtered through a 0.22 μm organic nylon membrane filter and transferred to a sample vial for subsequent UPLC-MS/MS analysis.

Chromatography–Mass Spectrometry Conditions

Chromatographic conditions: An Acquity UPLC BEH C_18_ column (2.1 mm × 100 mm, packing size 1.7 µm, Waters, Millford, MA, USA) was utilized. The column temperature was maintained at 40 °C; and the injection volume was set at 5 µL. The mobile phase consisted of 0.1% formic acid–2 mM ammonium acetate solution (phase A) and methanol (phase B), and the gradient elution program was set as shown in the Table 1.

Mass spectrometry conditions: Detection was performed using positive ion scanning under electrospray ionization in multiple reaction monitoring mode (cone voltage of 36 V; quantitative and qualitative ion pairs were 285.1 > 154.1 and 285.1 > 193.1, with collision voltages of 30 and 24 V, respectively). The capillary voltage was set at 3.5 kV, and the ionization source temperature was 120 °C. The desolvation gas temperature was 380 °C. Nitrogen (purity 99.9%) was used as the cone gas and the desolvation gas, while argon (purity 99.999%) was used as the collision gas. Cone gas and desolvation gas flow rates were 50 L/h and 600 L/h, respectively.

#### 2.3.4. Methodological Evaluation

Sample Matrix Effect Evaluation

The matrix effect (ME) refers to the inhibition or enhancement of ionization efficiency of the target analyte caused by non-target components of the sample (such as proteins, salts, and organic acids), which leads to a shift in the signal response of the MS. The ME was determined according to the methods in the literature and calculated using the following formula [32]:

$$\mathrm{ME}\;=\left(\frac{S_{m}}{S_{s}}-1\right)\times 100\%\;\;$$

where S_m_ and S_s_ represent the slopes of linear equations of matrix-matched calibration curves and solvent-configured standard curves, respectively. A positive ME indicated matrix enhancement, while a negative ME suggested matrix inhibition; |ME| ≤ 20% denotes a weak matrix effect, 20% ˂ |ME| ≤ 50% indicates a moderate matrix effect, and |ME| > 50% implies a strong matrix effect.

Linearity Range, Method Sensitivity, and Accuracy

The linear relationship and correlation coefficient between the intensity of the mass spectral response and the concentration of diazepam were examined after online analysis of the blank matrix-matched calibration curve solutions prepared according to Section 2.3.1. For method sensitivity evaluation, the concentration of the analyte corresponding to a mass spectrometry response with a signal-to-noise ratio (S/N) of 3 is defined as the limit of detection (LOD), and the concentration corresponding to an S/N of 10 is defined as the limit of quantitation (LOQ).

To evaluate the method’s accuracy and precision, diazepam solution was spiked into blank matrix samples of several representative aquatic products (negative samples that were tested to be free of diazepam) at concentrations of 0.5–5.0 μg/kg, followed by the addition of extraction solvent. Subsequent sample pre-treatment and instrumental analysis were performed according to Section 2.3.3. The ratio of the measured value to the theoretical value was used as the recovery to assess the accuracy of the method. The method reproducibility was evaluated based on the relative standard deviation (RSD) obtained from parallel determinations.

#### 2.3.5. Data Processing

All results are expressed as the means ± standard deviation of three or six independent experiments. Analysis of variance (ANOVA) was performed using SPSS 27.0 software (IBM Corporation, New York, NY, USA). *p*-values < 0.05 was considered statistically significant. Microsoft Excel 2010 (Microsoft Corporation, Washington, DC, USA) and Origin 8.0 (OriginLab Corporation, Northampton, MA, USA) were used to prepare graphs, and error bars on the graphs were generated from the standard deviation values.

## 3. Results and Discussion

### 3.1. Structural Characterization of Fe_3_O_4_@SiO_2_-PSA Magnetic NPs

#### 3.1.1. Material Morphology

**Figure 1:**
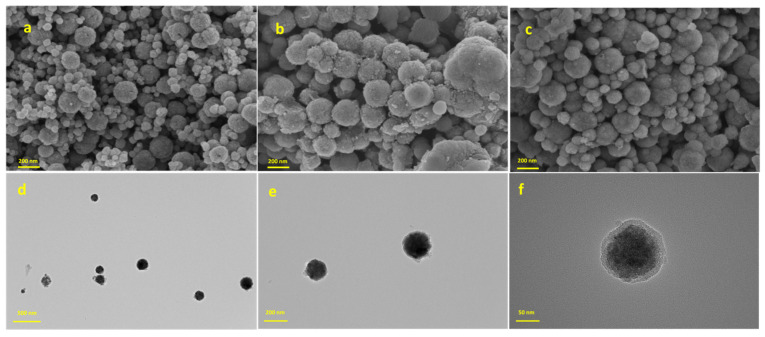
SEM images of Fe_3_O_4_ (**a**), Fe_3_O_4_@SiO_2_ (**b**), and Fe_3_O_4_@SiO_2_-PSA (**c**) and TEM images of Fe_3_O_4_@SiO_2_-PSA (**d**–**f**).

SEM was used to characterize and analyze the surface morphology of the synthesized microspheres. As shown in Figure 1a, the Fe_3_O_4_ nanoparticles exhibited a regular spherical structure (average particle size of about 200 nm). Due to magnetic interactions, the magnetic Fe_3_O_4_ NPs displayed obvious agglomeration and poor dispersion. Figure 1b,c demonstrate the morphological characteristics of nanomicrospheres Fe_3_O_4_@SiO_2_ and Fe_3_O_4_@SiO_2_-PSA, respectively. After silica shell coating and PSA modification, the average particle size of the Fe_3_O_4_@SiO_2_-PSA microspheres increased to about 245 nm, and the surface formed an apparent rough coating, a phenomenon that confirmed the successful construction of the core–shell structure.

The microstructure of the Fe_3_O_4_@SiO_2_-PSA NPs was further characterized using TEM. As shown in Figure 1d–f, the prepared Fe_3_O_4_@SiO_2_-PSA NPs maintained the spherical morphology. The high-resolution TEM images (Figure 1f) revealed that the Fe_3_O_4_ NPs are coated with a uniform SiO_2_ layer at the periphery. Notably, the nonmagnetic SiO_2_ capping layer effectively attenuated magnetic interactions between the magnetic cores of Fe_3_O_4_ [33], significantly improving NP dispersion and reducing the agglomeration phenomenon (Figure 1d).

#### 3.1.2. VSM

**Figure 2:**
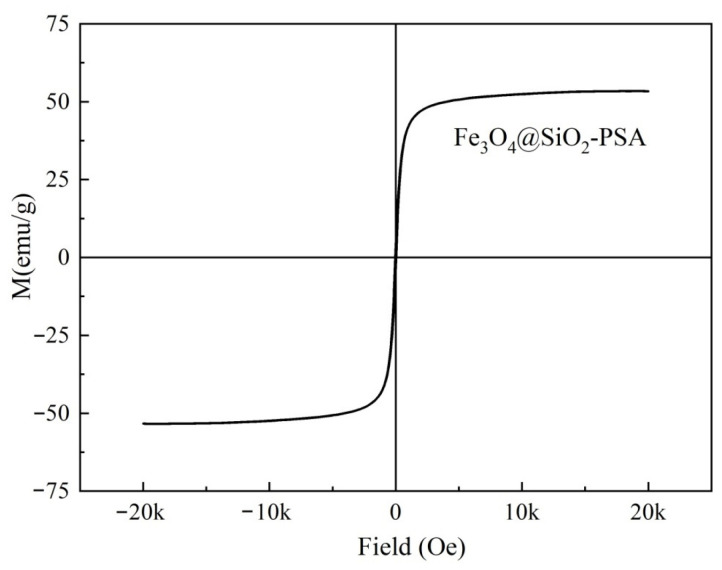
Magnetic hysteresis loop of Fe_3_O_4_@SiO_2_-PSA.

As shown in Figure 2, the hysteresis loop of Fe_3_O_4_@SiO_2_-PSA exhibited strong superparamagnetic properties at room temperature, with a saturation magnetization strength (*M_s_*) of 54.38 emu/g and a coercivity (*Hc*) close to zero. This result indicates that the material can display fast magnetic responsiveness under an applied magnetic field and avoid residual magnetic aggregation after the magnetic field is withdrawn, significantly improving its maneuverability and recyclability in adsorption–separation applications.

#### 3.1.3. XRD

**Figure 3:**
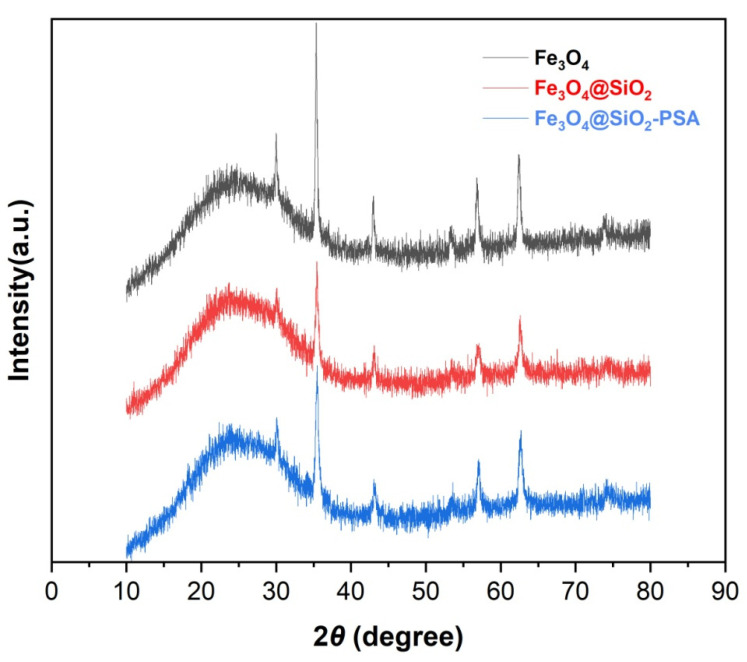
XRD patterns of Fe_3_O_4_, Fe_3_O_4_@SiO_2_, and Fe_3_O_4_@SiO_2_-PSA.

Figure 3 shows the XRD patterns of Fe_3_O_4_, Fe_3_O_4_@SiO_2_, and Fe_3_O_4_@SiO_2_-PSA NPs. The results showed that six characteristic diffraction peaks (2θ = 24.08°, 30.02°, 35.37°, 43.03°, 56.85°, and 62.48°) of Fe_3_O_4_ were observed in the range of 2θ = 10–80°, and the positions of these diffraction peaks coincided with the standard diffraction data of Fe_3_O_4_ (JCPDS No. 19-0629), confirming the successful preparation of Fe_3_O_4_ NPs with a spinel structure. Compared with Fe_3_O_4_, the Fe_3_O_4_@SiO_2_ composite showed a broadened diffraction peak near 2θ = 23.65°, which was attributed to the (100) crystalline surface of amorphous SiO_2_. This result confirmed that the SiO_2_ shell layer was successfully encapsulated on the Fe_3_O_4_ surface. In addition, the XRD patterns indicated that Fe_3_O_4_@SiO_2_ and Fe_3_O_4_@SiO_2_-PSA composites retained the characteristic diffraction peaks of Fe_3_O_4_ after SiO_2_ coating and PSA modification, suggesting that the crystal structure of Fe_3_O_4_ remained intact during the subsequent modification process. However, the intensity of the diffraction peaks of the composites was significantly weakened compared with that of pure Fe_3_O_4_, likely due to the encapsulation effect of the amorphous SiO_2_ shell layer, as well as the surface modification of the PSA molecules. Overall, the above results demonstrate the successful synthesis of Fe_3_O_4_@SiO_2_-PSA composites.

#### 3.1.4. FTIR

**Figure 4:**
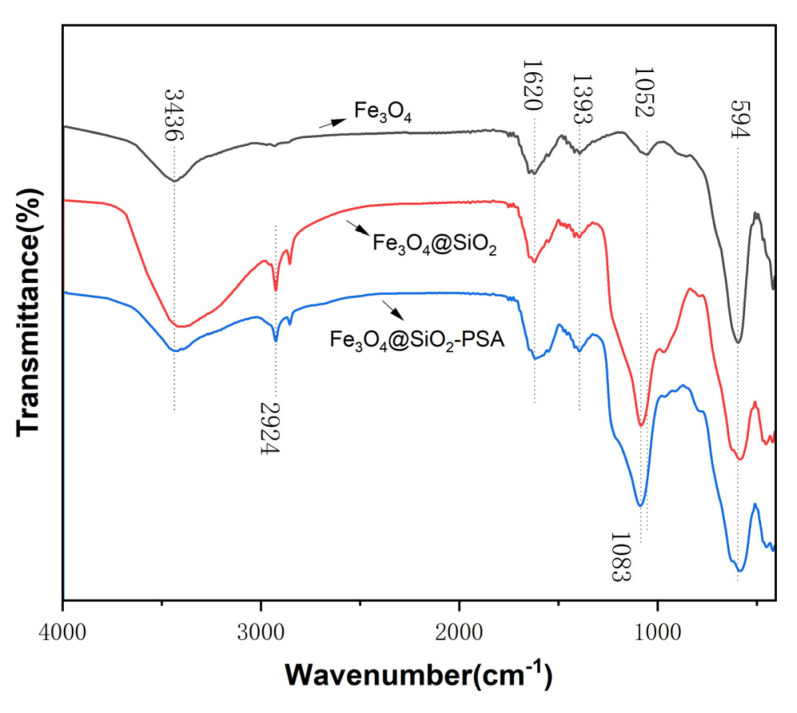
FTIR spectra of Fe_3_O_4_, Fe_3_O_4_@SiO_2_, and Fe_3_O_4_@SiO_2_-PSA.

Figure 4 demonstrates the FTIR spectra of three materials, Fe_3_O_4_, Fe_3_O_4_@SiO_2_, and Fe_3_O_4_@SiO_2_-PSA. The broad peak at 3436 cm^−1^ corresponded to the O-H stretching vibration of the hydroxyl group on the surface of Fe_3_O_4_. Comparison with the original spectra of Fe_3_O_4_ revealed that the SiO_2_-coated Fe_3_O_4_@SiO_2_ and its derivatives showed a significant Si-O-Si anti-symmetric telescopic vibration peak at 1083 cm^−1^. This characteristic signal confirms that silica has been successfully coated on the surface of Fe_3_O_4_. The C-H telescopic vibration peak of Fe_3_O_4_@SiO_2_ occurred at 2924 cm^−1^, forming a pair with the 2854 cm^−1^ peak, which is consistent with the C-H vibration characteristics of long-chain alkyl groups. Fe_3_O_4_@SiO_2_-PSA also showed a C-H stretching vibration peak at 2924 cm^−1^, attributed to the introduction of methylene groups (-CH_2_-) in the PSA molecule, which was chemically bonded to the SiO_2_ surface. This resulted in an increase in the number of organic chain segments. In addition, the characteristic Fe-O stretching vibration peaks of Fe_3_O_4_ were retained near 594 cm^−1^ for all three materials, with no significant shift in peak position or intensity. This result indicates that the crystal structure of Fe_3_O_4_ magnetic NPs remains intact during SiO_2_ coating and PSA modification, and the surface modification process does not change their intrinsic properties.

#### 3.1.5. BET Analysis

**Figure 5:**
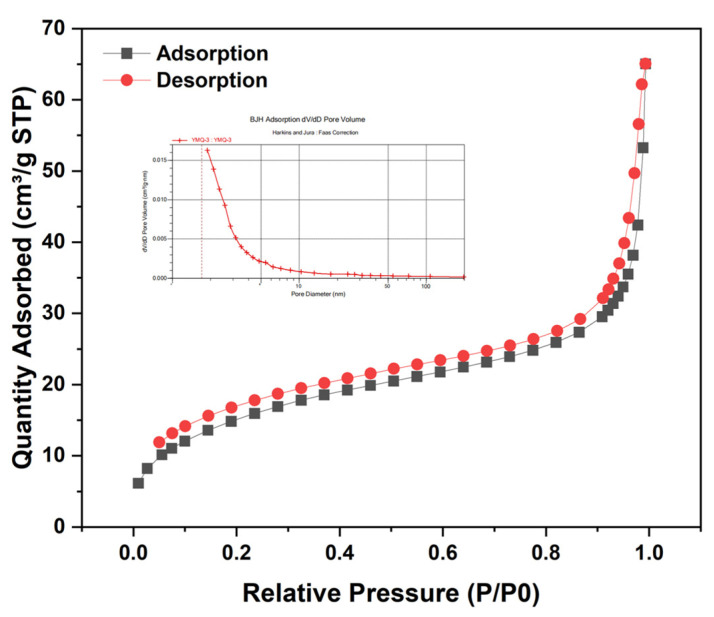
Nitrogen adsorption–desorption isothermal test curve and adsorption pore size distribution curve of Fe_3_O_4_@SiO_2_-PSA.

To characterize the mesoporous structural properties of Fe_3_O_4_@SiO_2_-PSA magnetic nanomaterials, the BET specific surface area and pore size distribution were fractionated using the nitrogen adsorption–desorption method. The results are shown in Figure 5. The BET specific surface area of the material was 57.12 m^2^/g, and the total pore volume of single-point adsorption was 0.101 cm^3^/g. Based on the calculations obtained using the BJH model, the average adsorption and desorption pore diameters were 7.04 nm and 4.25 nm, respectively. The nitrogen adsorption–desorption isotherm showed a type IV isotherm behavior (IUPAC classification), which was specifically expressed as follows: in the low relative pressure region (0 < P/P0 < 0.1), the adsorption increased to form an inflection point, indicating the existence of a monolayer adsorption behavior; with the increase in the relative pressure, the formation of a second layer began until the saturated vapor pressure was reached, at which point the number of adsorbed layers was infinite; when the relative pressure increased to the range of 0.4–0.8, there was a clear H1-type hysteresis loop, which was a typical capillary coalescence characteristic of mesoporous materials. However, the isotherms did not completely close in the low-pressure region, which might be related to the combined effect of irreversible chemisorption on the surface of the material and the microporous hysteresis effect. Lewis acidic sites on the Fe_3_O_4_ surface might produce strong adsorption of nitrogen, while organic functional groups introduced by PSA modification could lead to the formation of some sub-nanopore channels, resulting in a mesoporous capillary coalescence effect and requiring lower pressure to completely escape nitrogen during desorption.

### 3.2. Optimization of Dispersive Magnetic Solid-Phase Extraction Conditions

#### 3.2.1. Optimization of Extraction Solvent Type

**Figure 6:**
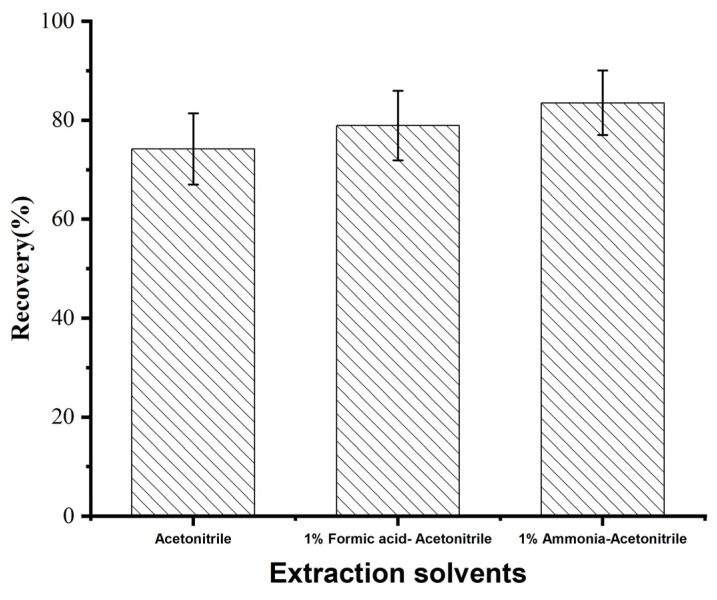
Effect of different extraction solvents on the recovery of diazepam.

Diazepam is a fat-soluble substance, and the main extraction solvents commonly used are acetonitrile [34,35] and ethyl acetate [36]. Considering that ethyl acetate is volatile, strongly irritating, and easy to emulsify when extracting samples with high levels of fat-soluble impurities [37], acetonitrile was selected as the extracting agent to reduce the background interference. To further investigate the effects of different extraction environments on extraction efficiency, this experiment compared the extraction performance of acetonitrile, 1.0% (*v*/*v*) formic acid in acetonitrile, and 1.0% (*v*/*v*) ammonia in acetonitrile as the sample extraction solvents with the blank *Carassius auratus* substrate spiked with 2.0 μg/kg diazepam to investigate the recoveries of the target compound. As shown in Figure 6, the highest recovery was obtained with 1.0% (*v*/*v*) ammonia in acetonitrile as the extraction solvent. Diazepam is a weakly basic drug with a dissociation constant pKa of about 3.3. In the alkaline environment (pH of around 10–11) provided by 1% ammonia, diazepam exists in its nonionic state, exist predominantly as a lipophilic free base, enhancing its solubility in acetonitrile. Meanwhile, the alkaline environment disrupts protein hydrogen bonds and salt bridges in aquatic matrices, releasing diazepam from binding sites and reducing adsorption loss [38]. Similarly, Zhang [39] et al. also concluded that for weakly basic analytical compounds, the mixed acetonitrile–ammonia system effectively improved extraction efficiency.

#### 3.2.2. Optimization of Volume of Extraction Solvent

**Figure 7:**
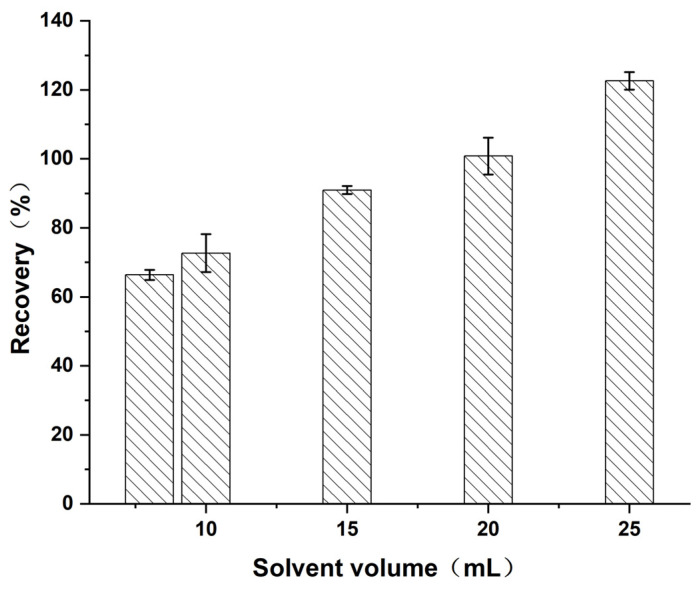
Effect of extraction solvent volume on the recovery of diazepam.

To determine the optimum extraction solvent volume, the extraction efficiency of 1.0% (*v*/*v*) ammonia acetonitrile solution at different volumes (8, 10, 15, 20, and 25 mL) was investigated in this study. The experimental results are shown in Figure 7. The recovery of diazepam significantly increased with increasing solvent volume (*p* < 0.05), which was attributed to the high solubility of diazepam in ammonia acetonitrile, and the increase in the solvent volume improved the dissolution efficiency of the target. However, when the volume of the extract was increased to 25 mL, the recovery exceeded 120%. The matrix of aquatic products is complex, and excessive solvent volume may lead to the dissolution of more fat-soluble impurities and increase the matrix effects, thereby influencing subsequent MS analysis. From the perspectives of economy and experimental efficiency, 15 mL was selected as the optimal extraction solvent volume, at which point the recovery of diazepam was 91.4%, ensuring high extraction efficiency and effectively reducing matrix interference.

#### 3.2.3. Optimization of MgSO_4_ Amount

**Figure 8:**
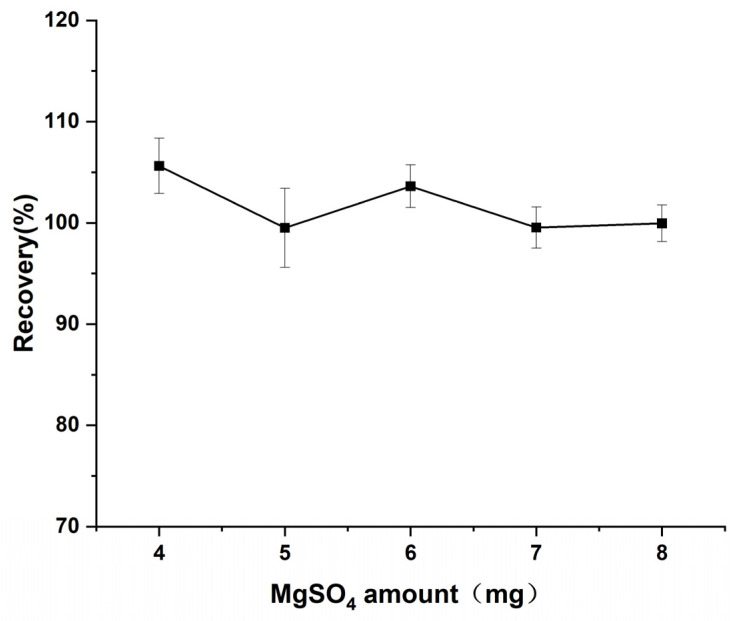
Effect of MgSO_4_ amount on the recovery of diazepam.

MgSO_4_ and NaCl are the core purification agents in the QuEChERS (quick, easy, cheap, effective, rugged, and safe) method for detecting drug residues in aquatic products. MgSO_4_ is responsible for the dehydration and adsorption of polar impurities, while NaCl separates the organic phase from the aqueous phase and inhibits the emulsification by salting out, which was combined to achieve better purification results [40]. In this study, the effect of different MgSO_4_ amounts (4–8 g) on the recovery of the target compound was systematically investigated, and the results are shown in Figure 8. When the amount of MgSO_4_ was 4 g, the recovery was slightly higher than 100% (106 ± 2.73%), suggesting the possibility of incomplete purification. At an amount of 5 g, the recovery tended to be in the desirable range (99.5 ± 3.87%), indicating that optimal purification was achieved. When the amount was further increased to 6–8 g, the recoveries fluctuated slightly and were not significantly different from those of the 5 g group (*p* > 0.05), indicating that the purification effect of MgSO_4_ had reached saturation. Based on the above results, 5 g was determined to be the optimal MgSO_4_ amount.

#### 3.2.4. Optimization of Fe_3_O_4_@SiO_2_-PSA Amount

**Figure 9:**
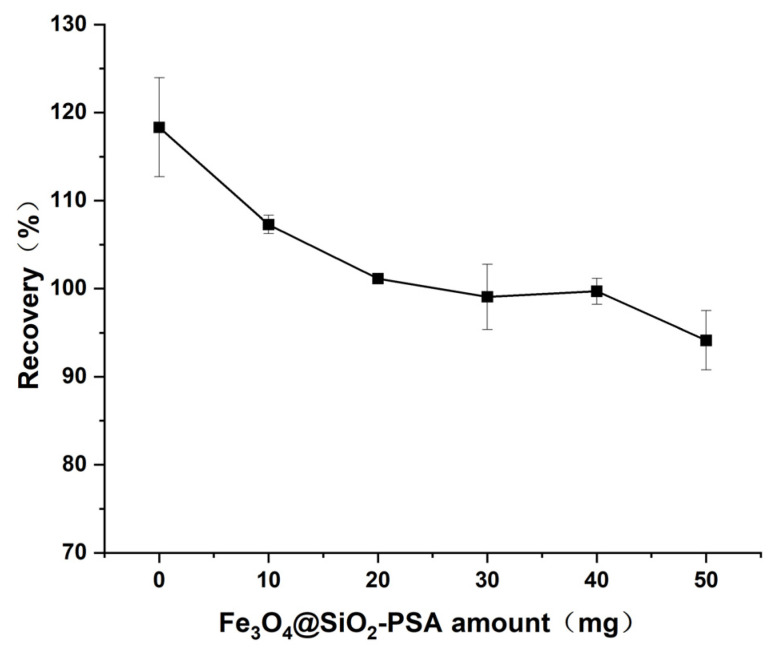
Effect of Fe_3_O_4_@SiO_2_-PSA amount on the recovery of diazepam.

PSA is a commonly used solid-phase dispersion purification material, which is widely used in antibiotic residue analysis due to its ability to effectively remove water-soluble impurities such as organic acids, fatty acids, and polar pigments as well as polar interferences such as sugars and fatty acids from the extract of biological samples [41,42,43]. Aquatic product matrices are rich in proteins and lipids, and during the extraction of target compounds, these co-extracts interfere with subsequent analysis and detection, affecting the determination of the results [44]. Therefore, establishing an effective purification method for sample pretreatment is crucial. In this study, the effects of Fe3O4@SiO_2_-PSA amount (0, 10, 20, 30, 40, and 50 mg) on the recovery of target additions were systematically investigated. As shown in Figure 9, one-way ANOVA showed that Fe_3_O_4_@SiO_2_-PSA amount significantly affected diazepam recovery (*p* < 0.05). When Fe_3_O_4_@SiO_2_-PSA was not added, the recovery was abnormally high due to a significant matrix effect. As the amount of Fe_3_O_4_@SiO_2_-PSA increased, the recovery gradually stabilized and reached 99.1% at 30 mg, and no significant change in recovery was observed with further increases in amount (*p* > 0.05). This indicates that 30 mg of Fe_3_O_4_@SiO_2_-PSA meets the purification requirements and achieves adsorption equilibrium. Therefore, 30 mg was chosen as the optimum amount of Fe_3_O_4_@SiO_2_-PSA.

#### 3.2.5. Optimization of C_18_ Amount

**Figure 10:**
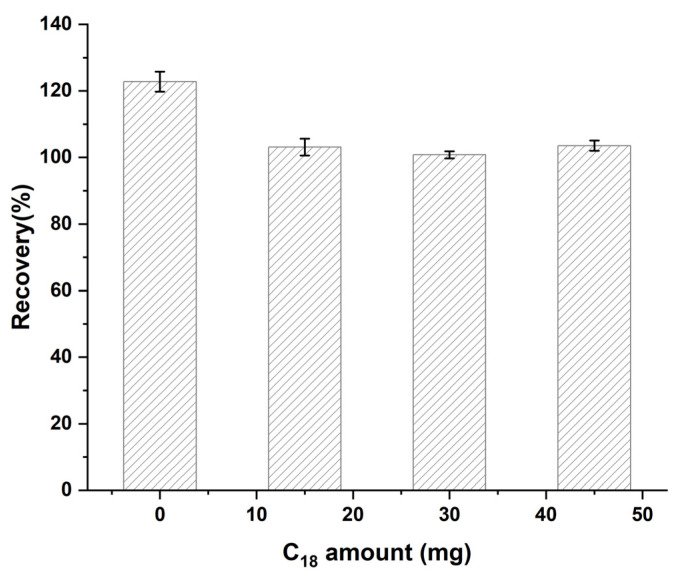
Effect of C_18_ amount on the recovery of diazepam.

As a commonly used filler for reversed-phase chromatography, C_18_ is often employed as a purification material in sample pretreatment because of its good adsorption properties for non-polar and weakly polar compounds, such as fats, sterols, and volatile oils [45]. In practical applications, C_18_ is typically used in combination with PSA to effectively remove various interfering substances such as lipids, sugars, and pigments from samples. In this study, the effect of C_18_ addition (0, 15, 30, and 45 mg) on diazepam spiking recovery was investigated. The results are shown in Figure 10. When no C_18_ was used for purification, the sample matrix caused severe interference, resulting in a significantly high recovery of the target (123%). With the addition of 15–45 mg of C_18_, the recoveries significantly improved and stabilized within the range of 101–104%, which was in line with the requirements for residue analysis. When the amount of C_18_ was increased to 30 mg, the recovery stabilized (101%); thus, 30 mg was finally chosen as the optimal amount.

#### 3.2.6. Optimization of Adsorption Shaking Time

**Figure 11:**
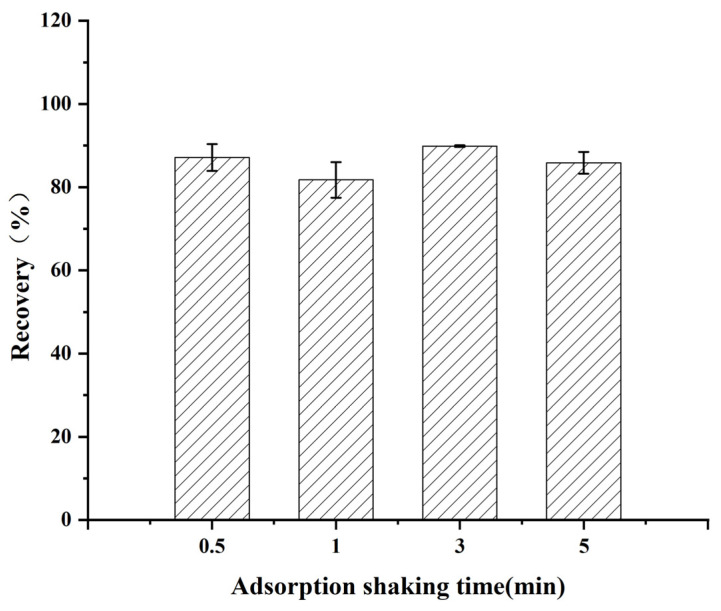
Effect of adsorption shaking time on the recovery of diazepam.

Adsorption shaking time is also one of the key parameters in the DSPE process, which directly affects the mass transfer efficiency between analytes and adsorbents. As shown in Figure 11, the shaking time significantly affected the cleanup effect. When the shaking time was 3 min, the target recovery reached its peak (89.9%), indicating that the optimal balance between the efficient release of the target and the effective adsorption of impurities could be achieved under this condition. Insufficient shaking time (0.5–1 min) leads to incomplete desorption of the target or inadequate adsorption of the impurities, which can lower the recovery, while a long shaking time (5 min) may cause thermal degradation of the target or enhance nonspecific adsorption. Based on the above results, the optimal adsorption shaking time was finally determined to be 3 min.

### 3.3. Methodological Evaluation

#### 3.3.1. Matrix Effects

**Figure 12:**
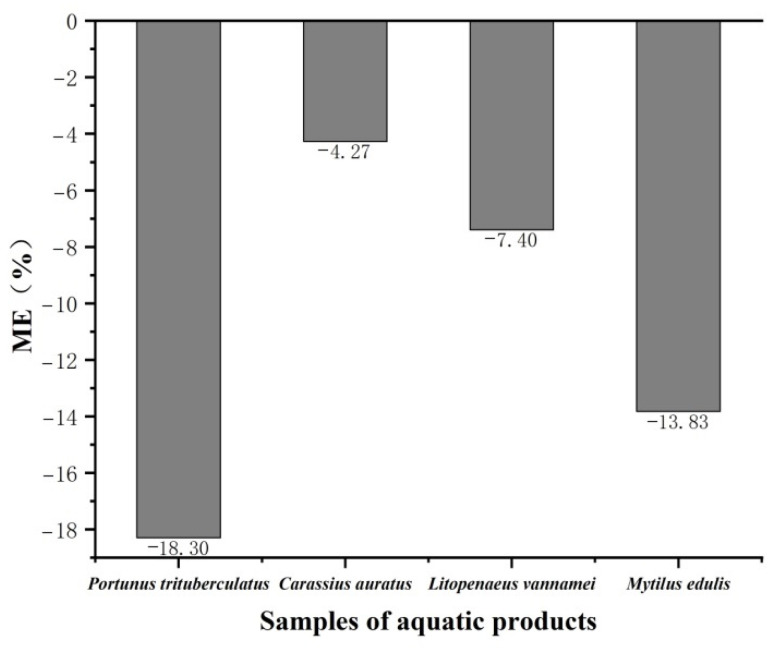
Matrix effects of diazepam in different aquatic products.

Incomplete purification of impurities during sample pretreatment can trigger matrix effects, leading to enhancement or suppression of analyte signals, thus affecting the accuracy of measurement results. Therefore, matrix effects must be evaluated during method validation. In this study, matrix effects were evaluated in aquatic products such as *Carassius auratus*, *Litopenaeus vannamei*, *Portunus trituberculatus*, and *Mytilus edulis* by comparing the slopes of matrix-matched calibration curves with those of solvent calibration curves. The results, as shown in Figure 12, indicated that the matrix effects of diazepam in the four aquatic products ranged from −18.3% to −4.27%, demonstrating weak matrix inhibitory effects in all cases. Although existing methods often use expensive diazepam deuterium substitutes as internal standards to correct matrix interference [46], the present method achieved good purification by optimizing the pretreatment process, resulting in low matrix interference. Thus, it ensured the accuracy of the determination without the use of internal standards, significantly reducing the cost of the assay.

#### 3.3.2. Linear Range of Method, LOD, and LOQ

**Figure 13:**
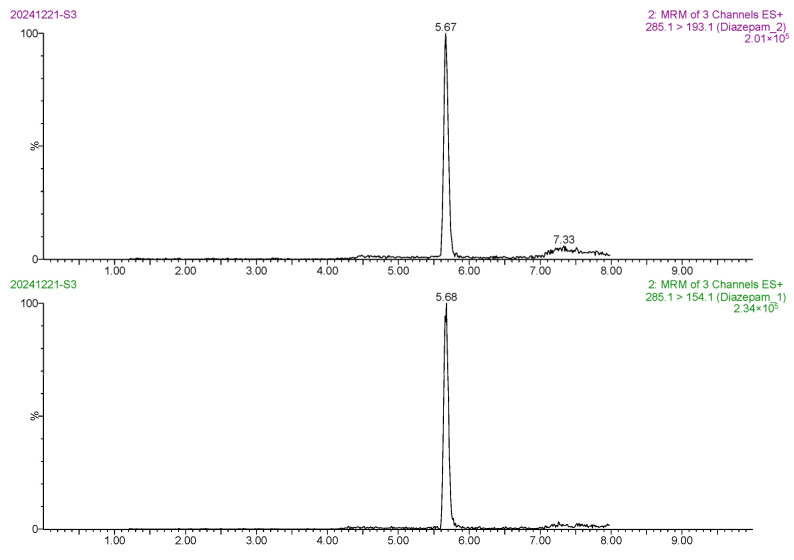
MRM chromatogram of 0.5 ng/mL diazepam standard solution.

**Figure 14:**
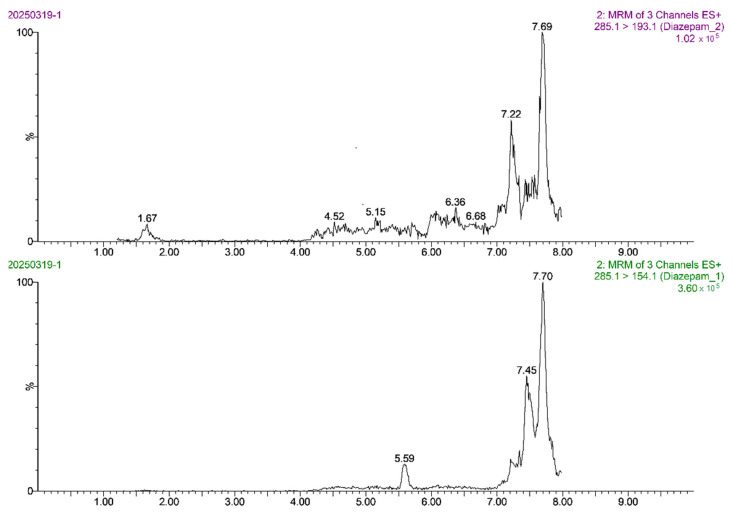
MRM chromatogram of diazepam determination in blank *Carassius auratus* matrix.

**Figure 15:**
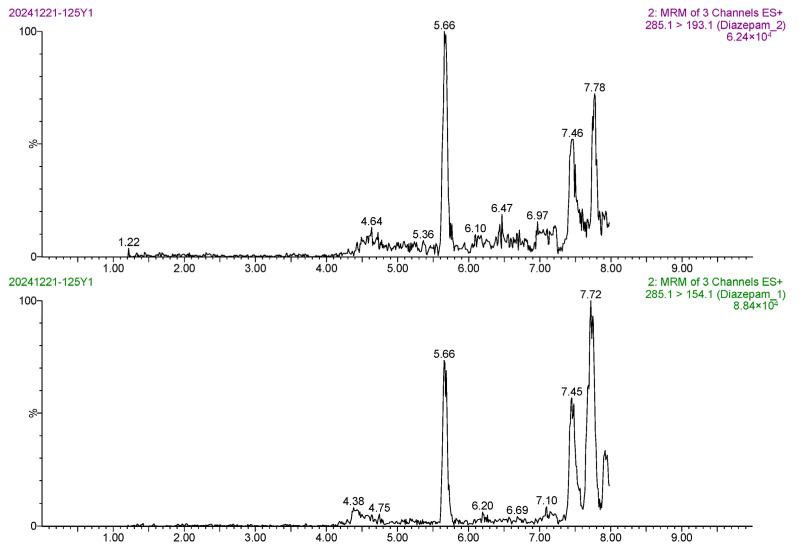
MRM chromatogram of diazepam determination at LOD spiked level in blank *Carassius auratus* matrix.

**Figure 16:**
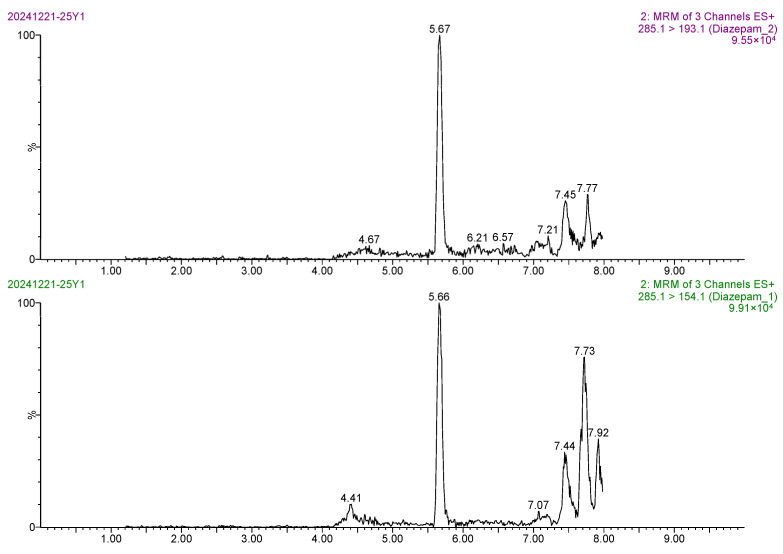
MRM chromatogram of diazepam determination at LOQ spiked level in blank *Carassius auratus* matrix.

The linear range of diazepam was 0.1–10 μg/L. The calibration curve of diazepam was linear between its mass spectral peak area (*y*) and its mass concentration (*x*), and the regression equation was *y* = 45,864.4*x* ± 4024.07. The correlation coefficient (*r*) was 0.9992, and the coefficient of determination (*r*^2^) was 0.9983. Based on the 3-fold and 10-fold signal-to-noise ratio (S/N), the LOD and LOQ of the method were 0.20 μg/kg and 0.50 μg/kg, respectively, indicating the high sensitivity of the method. The MRM chromatogram of the 0.5 ng/mL diazepam standard solution (Figure 13) showed sharp target peaks and strong MS responses. The MRM chromatogram of the blank *Carassius auratus* sample (Figure 14) showed no interfering peak at 5.6 min, indicating excellent method selectivity. In addition, the MRM chromatograms of the blank *Carassius auratus* matrix spiked at LOD and LOQ levels (Figure 15 and Figure 16) further verified the sensitivity of the method.

#### 3.3.3. Method Accuracy and Precision

The method was used to perform spiked recovery tests on four blank matrix samples of *Carassius auratus*, *Litopenaeus vannamei*, *Portunus trituberculatus*, and *Mytilus edulis* at spiked levels of 0.5, 1.5, 6.0, and 15.0 μg/kg, respectively. Six parallel tests were set up at each spiked level, and the average recoveries and relative standard deviations (RSDs) were calculated. As shown in Table 2, the average recoveries of diazepam in the spiked range of 0.5–15.0 μg/kg were from 74.9% to 109%, and the RSDs ranged from 1.24% to 11.6%, indicating that this method was accurate and precise, and suitable for analyzing and detecting diazepam residues in aquatic products.

The method was compared with the methods previously reported in the literature for the determination of diazepam residues in aquatic products, and the results are shown in Table 3. This method is superior to some of the existing methods in terms of accuracy and sensitivity, and it has the following significant advantages: First, MDSPE was used for the pretreatment of the sample, and the clean-up process could be completed by a single solvent extraction and centrifugation operation. Compared with the traditional QuEChERS method, this method does not require nitrogen blowing and concentration steps, and the pretreatment time of a single sample can be controlled within 30 min, thereby significantly improving analytical efficiency. Second, the method introduces less matrix interference during the pretreatment process, and satisfactory analytical results can be obtained using the external standard curve method without relying on expensive reagents such as a deuterium internal standard, thus significantly reducing the cost of the assay. The limitation of this study was the higher LOD (0.20 μg/kg) and LOQ (0.50 μg/kg) compared to some established methods. For instance, the approach described in Reference 47 achieved significantly lower detection limits, with an LOD range of 0.03–0.08 μg/kg and LOQ range of 0.10–0.24 μg/kg. This enhanced sensitivity gave the referenced method a distinct advantage in monitoring trace-level diazepam residues.

### 3.4. Actual Sample Validation

The method was utilized to determine the diazepam content in the fish meal matrix QC sample No. MRM1336 (China CFAPA Testing Technology Co., Ltd. of Dalian, China). The results of three independent determinations showed that the average diazepam content was 10.5 μg/kg (RSD = 4.78%, n = 3). Referring to the certified certificate of analysis for this QC sample, the standard value was 10.8 ± 2.2 μg/kg (k = 2). The absolute deviation of the test result from the standard value was 0.3 μg/kg, which was within the extended uncertainty range of the standard value, indicating that the determination system exhibited good accuracy. The method was further applied to conduct diazepam residue screening in 30 cultured fish samples (covering five species, including *Carassius auratus*, *Sciaenops ocellatus*, *Acanthopagrus schlegelii*, *Lateolabrax japonicas*, and *Larimichthys crocea*) collected from Zhoushan aquaculture farms in Zhejiang Province. Among them, diazepam was detected in one sample of *Carassius auratus* collected from a recreational fishing site, with a quantitative result of 2.25 μg/kg. The validation result of the quality control sample and the testing data of actual samples confirmed the reliability of the method for the trace detection of diazepam in complex aquatic matrices.

## 4. Conclusions

This study introduces a novel method for detecting diazepam residues in aquatic products by integrating MDSPE with UPLC-MS/MS, utilizing Fe_3_O_4_@SiO_2_-PSA core-shell magnetic nanomaterials. The synthesized material exhibited exceptional adsorption capacity and magnetic responsiveness, enabling efficient sample purification while outperforming traditional QuEChERS in simplicity, sensitivity, accuracy, and precision. This approach provides a robust analytical tool for monitoring diazepam residues in aquatic products, contributing to enhanced consumer safety.

Nevertheless, the study is subject to certain limitations. The optimization of MDSPE parameters, including extraction solvent type and solvent volume, relied solely on single-factor experiments, which may fail to account for potential interactions between variables. Future improvements could incorporate multifactorial optimization techniques (e.g., Box–Behnken or central composite design) to enable statistically validated optimization with higher efficiency and reliability and offer a robust methodological framework [52,53]. Additionally, the current method targets only diazepam; its extension to other benzodiazepines requires further validation.

Building on this foundation, future research could be directed toward the following avenues: (1) expanding the method’s application scope, adapting Fe_3_O_4_@SiO_2_-PSA for simultaneous extraction and detection of multiple benzodiazepines or other veterinary drugs; (2) enhancing nanoparticle performance, optimizing synthesis and functionalization (e.g., alternative coatings or ligands) to improve adsorption capacity, selectivity, and sensitivity for diazepam and related compounds.

## Figures and Tables

**Table 1:** Gradient elution conditions for mobile phase.

Time (min)	Flow (mL/min)	A (%)	B (%)
1	0.3	90.0	10.0
3	0.3	90.0	10.0
3.5	0.3	85.0	15.0
6	0.3	32.0	68.0
6.2	0.3	5.0	95.0
7.8	0.3	5.0	95.0
8	0.3	90.0	10.0
10	0.3	90.0	10.0

**Table 2:** Recovery and precision of diazepam in spiked samples of four aquatic product matrices.

Sample Type	Addition Level (μg/kg)	Recovery (%)	RSD (%, n = 6)
*Carassius auratus*	0.5	94.0	10.6
	1.5	89.8	5.17
	6.0	99.8	1.24
	15.0	108	11.6
*Litopenaeus vannamei*	0.5	76.9	4.19
	1.5	97.9	2.77
	6.0	99.9	6.64
	15.0	109	6.80
*Portunus trituberculatus*	0.5	74.9	4.96
	1.5	89.7	3.49
	6.0	108	2.93
	15.0	109	3.58
*Mytilus edulis*	0.5	107	6.03
	1.5	93.1	5.02
	6.0	100	9.93
	15.0	105	2.71

**Table 3:** Method comparison for diazepam residue analysis in aquatic products.

Method of Detection	Matrix	Purification	Quantification	Recoveries (%)	Precision (% RSD)	LOD (μg/kg)	LOQ (μg/kg)	Literature
UPLC-MS/MS	*Carps*, *Grass carp*, *Hypophthalmichthys nobilis*, *Tilapia*, *Crucian Carp*, *Turbot*, *Shrimp*, *Hypophthalmichthys molitrix*, *Catfish and Mussel*	Self-assembled SPE	Internal standard curve	81.6–113	0.9–7.5	0.03–0.08	0.10–0.24	[47]
LC-MS/MS	Fish and shrimp	C_18_ SPE	Matrix-matched calibration curves	77.94–104.27	1.43	0.01	0.03	[34]
LC-MS/MS	*Anguilla anguilla*	HLB Oasis SPE and Captiva EMR-lipid SPE	Internal calibration	82	1–4	0.22	0.75	[48]
UPLC-MS/MS	Freshwater fish	QuEChERS	Internal standard curve	96.1–97.6	2.5–4	0.53	1.76	[49]
UPLC-MS/MS	Fish	C_18_/PSA SPE	Internal standard curve	109.2–120	4.5–8.1	0.2	/	[50]
HPLC-ESI-MS/MS	*Carp*	QuEChERS (PSA, MWCNTs)	Matrix-match calibration curves external calibration curve	96–108.8	<10	0.5	2.5	[51]
UPLC-MS/MS	*Carassius auratus* *Solenoceracrassicornis* *Portunus trituberculatus* *Mytilus edulis*	MDSPE	External calibration curve	74.9–109	1.24–11.6	0.20	0.5	Present method

## Data Availability

The original contributions presented in the study are included in the article, further inquiries can be directed to the corresponding author.
